# Magnetic Properties of Electrodeposited Cobalt-Platinum (CoPt) and Cobalt-Platinum-Phosphide (CoPtP) Thin Films

**DOI:** 10.3389/fchem.2021.733383

**Published:** 2021-09-10

**Authors:** D.-Y. Park, N. V. Myung

**Affiliations:** ^1^ Department of Advanced Materials Engineering, Hanbat National University, Daejeon, South Korea; ^2^ Department of Chemical and Biomolecular Engineering, University of Notre Dame, Notre Dame, IN, United States

**Keywords:** cobalt-platinum, cobalt-platinum-phosphide, electrodeposition, magnetic thin film, hard magnetic material

## Abstract

CoPt and CoPtP thin films were synthesized using direct current (DC) aqueous electrodeposition from weak alkaline solutions. The basic plating solutions of binary CoPt thin films consisted of cobalt pyrophosphate [Co_2_P_2_O_7_] and chloroplatinic acid [H_2_PtCl_6_]. Various amounts of sodium hypophosphite [NaH_2_PO_2_] was added to deposit ternary CoPtP thin films. The film composition was adjusted by varying the several electrodeposition parameters including electrolyte composition, solution pH, and current density and correlated to their microstructure and magnetic property (*i.e.* coercivity and squareness). For the binary CoPt thin films, the maximum coercivities [in-plane coercivity (H_c,//_) = ∼1,600 Oe, and perpendicular coercivity (H_c,⊥_) = ∼2,500 Oe] were obtained from electrolytes containing 0.01 M H_2_PtCl_6_ + 0.04 M Co_2_P_2_O_7_ at current density (CD) of 7.5 mA cm^−2^. In the case of ternary CoPtP electrodeposits, the maximum coercivities (H_c,//_ = ∼2,600 Oe, and H_c,⊥_ = ∼3,800 Oe) were achieved from baths containing 0.015 M H_2_PtCl_6_, 0.07 M Co_2_P_2_O_7_, 0.8 M NaH_2_PO_2_ at CD of 7.5 mA cm^−^^2^ and solution pH 9. It was suggested that microstructure and magnetic properties are affected not only by the type of substrate but also by chemical compositions and electrodeposition conditions.

## Introduction

CoPt and CoPtP alloys are promising hard magnetic materials due to their high magnetocrystalline anisotropy and magnetic saturation (Bozorth, 1963; Myung et al., 2003). Co_50_Pt_50_ alloy has tetragonal L1_O_ ordered phase material and shows very high coercivities (>10,000 Oe) (Coffey et al., 1995). Because of their excellent hard magnetic properties, they are of interest in the areas such as magnetic sensors and magnetic microelectromechanical systems (mag-MEMS) (Myung et al., 2003; Park et al., 1995; Vieux-Rochaz et al., 2006).

CoPt thin films were mostly obtained using vacuum processes such as molecular-beam epitaxy (MBE) (Lee et al., 1991), and sputtering (Coffey et al., 1995; Carcia et al., 1993; Farrow and Marks, 1998). In these vacuum processes, CoPt was deposited as multilayered structures and followed by post thermal treatment to make ordered phases. The requirement of post thermal treatment limited the applications including mag-MEMS since most of MEMS structure cannot survive at these high temperatures (e.g., 500–700 °C). Therefore, an alternative near room temperature deposition process such as electrodeposition is needed. Electrodeposition process over vacuum processes has many benefits such as easy scale up and maintenance, lower operating temperature, low cost, the ability of tailoring microstructure and properties. Therefore, it was widely used in many research fields including thin film, nanostructures including nanocrystals, nanorod etc. (Park et al., 1995; Myung et al, 2003; Zhu et al., 2015a; Zhu et al., 2015b).

Despite the needs for integration of magnetic CoPt and CoPtP alloys by electrodeposition at near room temperature, limited works were carried out for electrodeposition baths and conditions. Tabakovic et al. and Dragos-Pinzaru et al. conducted electroanalytical study and electroplating parameters (e.g., electrodeposition time, Co^2+^ concentration, additive, solution pH etc.) on film composition and magnetic properties (Tabakovic et al., 2015; Tabakovic et al., 2016; Dragos-Pinzaru et al., 2017). Kim et al. also investigated magnetic properties (*e.g.,* coercivity, magnetic moment etc.) to film thickness (Kim et al., 2013). Guillamat et al. electrodeposited CoPt thin film from deep eutectic solvent (Guillamat et al., 2012) whereas Hnida et al. electrodeposited nanowires using template directed method (Hnida et al., 2016). Eagleton et al. reported coercivity of 2,000–4,000 Oe for 50 nm–10 μm thick CoPtP films (Eagleton et al., 2005). Vieux-Rochaz et al. integrated hard magnetic CoPtP material into mag-MEMS (Vieux-Rochaz et al., 2006).

In this study, magnetic CoPt and CoPtP thin film alloys were systematically electrodeposited using the various plating solutions containing chloroplatinic acid, cobalt pyrophosphate and sodium hypophosphite. Dependence of various electrodeposition parameters including solution composition (e.g [Pt^4+^] [Co^2+^] and [H_2_PO_2_^−^]), solution pH, current density on current efficiency, magnetic properties, and microstructure were investigated.

## Experimental

**FIGURE 1:**
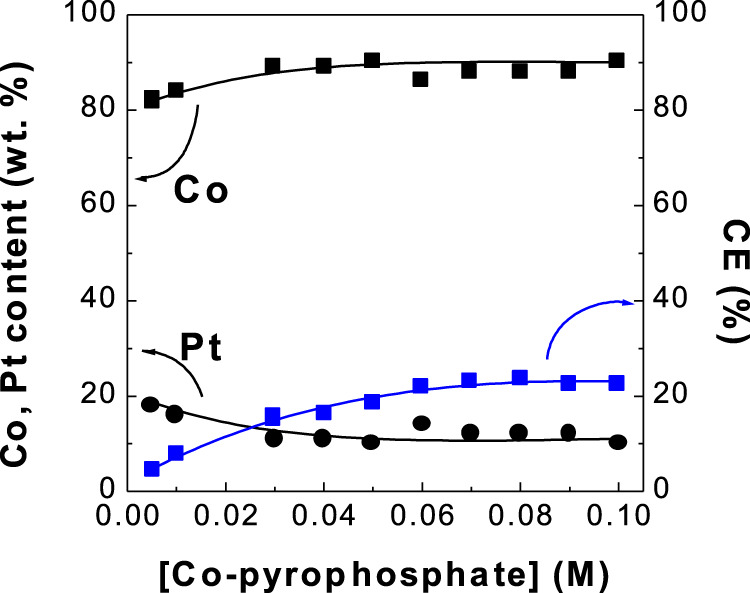
Dependence of Co, Pt contents and current efficiency (CE) of CoPt thin film on Co-pyrophosphate concentration.

**FIGURE 2:**
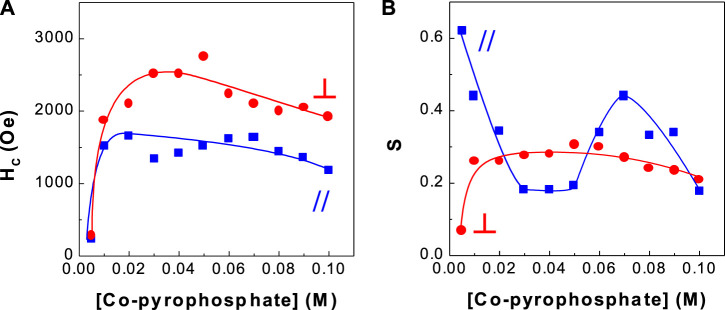
Dependence of coercivity and squareness of CoPt thin films on Co-pyrophosphate concentration **(A)** coercivity and **(B)** squareness.

**FIGURE 3:**
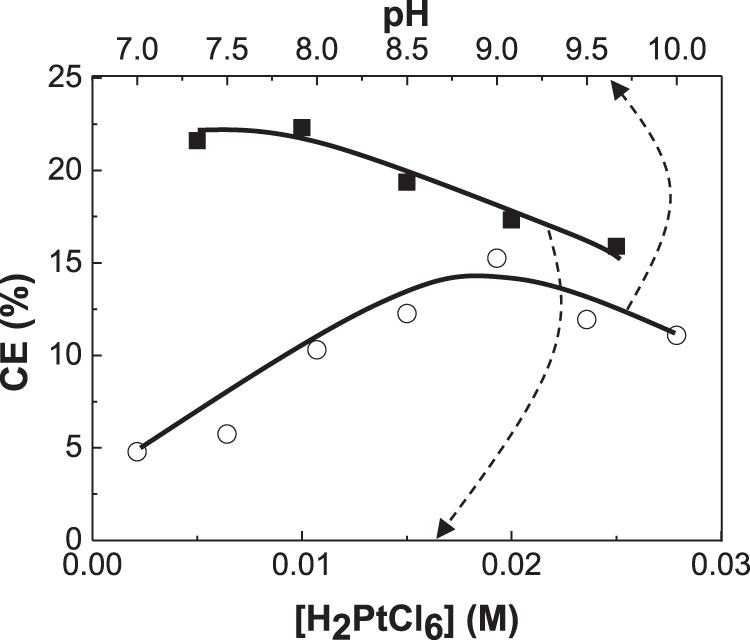
Dependence of current efficiency (CE) of CoPt thin films on H_2_PtCl_6_ concentration and solution pH.

**FIGURE 4:**
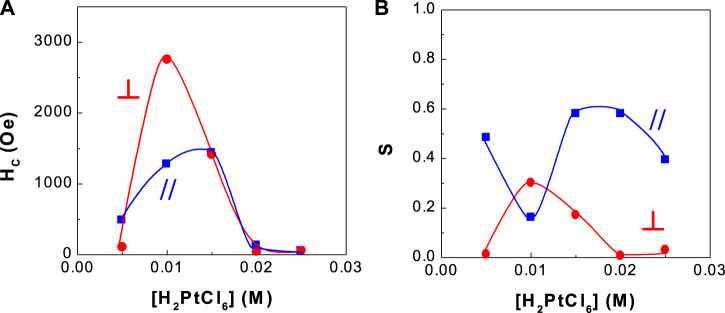
Dependence of coercivity and squareness of CoPt thin films on H_2_PtCl_6_ concentration **(A)** coercivity and **(B)** squareness.

**FIGURE 5:**
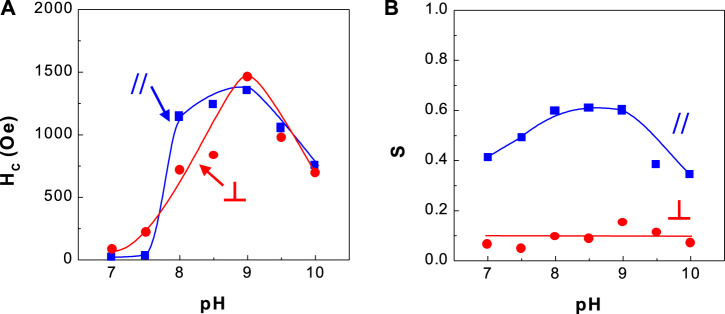
Dependence of coercivity and squareness of CoPt thin films and solution pH **(A)** coercivity and **(B)** squareness.

**FIGURE 6:**
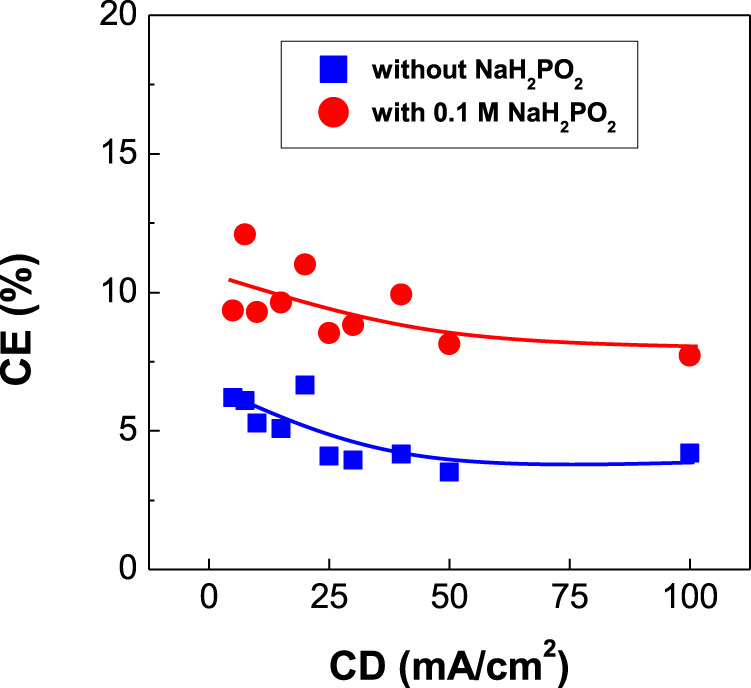
Dependence of current efficiency (CE) of CoPt thin films on current density (CD) (The values of CE in the red curve are similar or lower than that in Figures 1, 3).

**FIGURE 7:**
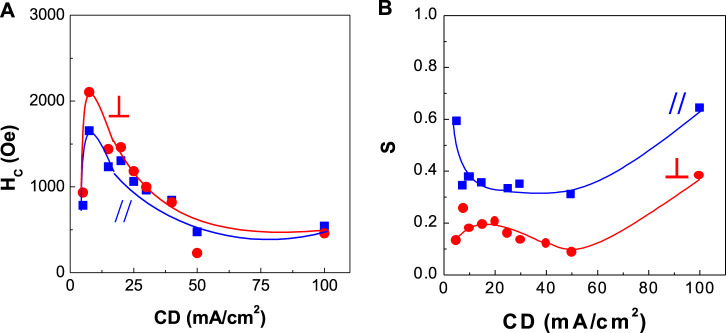
Dependence of coercivity and squareness of CoPt thin films on current density (CD) **(A)** coercivity and **(B)** squareness.

**FIGURE 8:**
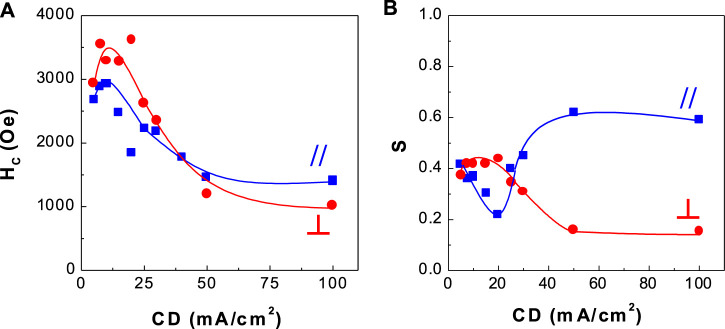
Dependence of coercivity and squareness of CoPtP thin films on current density (CD) **(A)** coercivity and **(B)** squareness.

**FIGURE 9:**
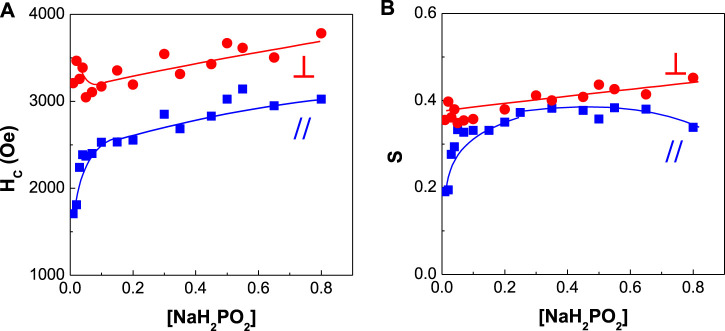
Dependence of coercivity and squareness of CoPtP thin films on NaH_2_PO_2_ concentration **(A)** coercivity and **(B)** squareness.

For binary CoPt thin films, the dependence of deposit contents, current efficiencies, and extrinsic magnetic properties (*i.e.,* coercivity and squareness) on Co^2+^ ion concentration in plating baths was investigated. The bath compositions and operating conditions are listed in Table 1 (conditions for Figures 1,2). Co^2+^ concentration in the bath was controlled using Co-pyrophosphate solution as shown in Table 2 (conditions for Figures 1,2). After the optimum Co^2+^ concentration in the bath for the best coercivity (H_c,⊥_ and H_c,//_) of CoPt thin film was determined, the dependence of current efficiency and coercivity in CoPt thin films on concentration of Pt^4+^ in plating bath was studied. The bath compositions and operating conditions are listed in Table 1 (conditions for Figures 3,4). Both Co^2+^ and Pt^4+^ concentrations for the optimum coercivity of CoPt thin film were determined as 0.07 and 0.015 M, respectively and listed in Table 1 (conditions for Figures 3,5). Dependence of current efficiencies in the CoPt thin films on solution pH was also studied. Then a better bath composition and solution pH (conditions for Figures 6,7,8) was determined as shown in Table 1. The optimum current density for the best coercivity of CoPt thin film was tested using the bath compositions and conditions of Table 1 with/without NaH_2_PO_2_ concentration (as P source). Finally, the optimum electroplating conditions for the best coercivity of CoPt thin film as shown in Table 1 (conditions for Figure 9) was obtained. Magnetic properties [parallel (in-plane) and perpendicular (out-of-plane) coercivity (H_c,//_ and H_c,⊥_), and parallel (in-plane) and perpendicular (out-of-plane) squareness (S_//_ and S_⊥_)] were examined by varying the NaH_2_PO_2_ concentration from 0.01 to 0.8 M. All the CoPt and CoPtP thin films were electrodeposited on brass substrates; Pt (mesh) coated on Ti core was used as an insoluble anode. Brass substrates were used because they exhibit no magnetic property, specially coercivity and squareness, at all. Solutions were exposed to air. All the films were electrodeposited without stirring at room temperature.

**TABLE 1:** Bath compositions and operating conditions (unless otherwise noted) for binary CoPt thin films (M = mol dm^−3^).

Chemical/condition	Concentration/unit				
Pt^4+^ (as H_2_PtCl_6_∙6H_2_O) (M)	0.01	0.005–0.025	0.015	0.01	0.015
Co^2+^ (as Co_2_P_2_O_7_ referred to Table 2) (M)	0.005–0.1	0.07	0.07	0.02	0.07
Na_3_PO_4_∙12H_2_O (M)	0.365	0.365	0.365	0.365	0.365
NaH_2_PO_2_∙H_2_O (M)	—	—	—	0 or 0.1	0.01–0.8
Solution pH	8	8	7–10	8	9
Current density (mA∙cm^−2^)	7.5	7.5	7.5–100	7.5–100	7.5
Deposit charge (C) (deposit time) (sec)	15 (2,000)	15 (2,000)	15 (2,000)	15 (2,000)	15 (2,000)
Corresponding figures	1 and 2	3 and 4	3 and 5	6, 7 and 8	9

**TABLE 2:** Bath compositions (unless otherwise noted) for Co-pyrophosphate solution (M = mol dm^−3^).

Chemical/condition	Concentration (M)/unit
Co^2+^ (as CoSO_4_∙7H_2_O)	0.120 M
Na_4_P_2_O_7_	0.451 M
NH_4_OH	1 ml/L
Solution pH	8.5

Deposit Co and Pt contents in CoPt and CoPtP thin films were analyzed using atomic absorption spectroscopy (AAS). P content in the CoPtP thin films could not be analyzed using both AAS and energy dispersive spectroscopy (EDS) because of interference between Pt and P elements. Magnetic properties such as coercivity (H_C_) and squareness (S = M_r_/M_S_) were measured using a vibrating sample magnetometer (VSM) (Model 880, ADE technologies Inc.). Microstructures of CoPt and CoPtP thin films were examined using an X-ray diffractometer (XRD) (Model 42202, Norelco, North American Phillips Company Inc.) with K_α_ radiation to identify the phases. Conditions of XRD were a scanning range of 20–100° with 0.03° increments and a one second dwell time.

## Results and Discussion

Figure 1 shows the dependence of Co and Pt contents in electrodeposits and current efficiency (CE) on Co^2+^ concentrations. Deposited Co content in electrodeposits increased from 82 to 90 wt% with increasing Co^2+^ concentration from 0.005 to 0.1 M, while Pt content decreased from 19 to 10 wt%. Current efficiency increased from 4 to 22%. Very limited work for deposit Co content and current efficiency in CoPt alloy was reported. Dragos-Pinzaru et al. electrodeposited CoPt films from hexachloroplatinate solutions: 0.4 M H_3_BO_3_, 0.3 M NH_4_Cl, 0.1 M CoSO_4_∙7H_2_O, 0.00386 M H_2_PtCl_6_ with/without 3.89 mM saccharin (Dragos-Pinzaru et al., 2017). They reported deposit Co content of 28.8–66.9 wt% (56–87 at%) with the change of deposit time from 10 s to 300 s. Deposit Co content of 82–90 wt% in this study is significantly higher than that (28.8–66.9 wt%) in the article reported by Dragos-Pinzaru et al. (Dragos-Pinzaru et al., 2017). Also, they investigated the effect of deposit time on current efficiency at different pH and different Co^+2^ ion concentration and reported the current efficiency of 55–68% and 55–78% with the change of deposit time, respectively. They reported about 2.5–20 times higher current efficiency (55–78%) than that (4–22%) of this study. Relatively low current efficiency in this study compared to that reported by Dragos-Pinzaru et al. (Dragos-Pinzaru et al., 2017) may be attributed to the different chemical compositions. That is, the applied current in this paper was used very much in side reactions such as the evolution of hydrogen gases on cathode and oxygen gases on anode.

Figure 2 shows the dependence of hard magnetic properties (*i.e.,* coercivity and squareness) of binary CoPt electrodeposits. The optimum Co^2+^ concentrations with high coercivity were observed in the range of 0.03–0.07 M. In this range of Co^2+^ concentrations, parallel coercivities ranged from ∼1,400 to ∼1,600 Oe whereas perpendicular coercivities ranged from ∼2,200 to ∼2,800 Oe. On the other hand, parallel and perpendicular squareness (S_//_ and S_⊥_) were measured to be ranged from ∼0.2 to ∼0.5 and about 0.25, respectively. Tabakovic et al. obtained the coercivity of H_C,//_ = 221 Oe and H_C,⊥_ = 254 Oe from Co_80_Pt_20_ films deposited on Cu substrate [oxidized Si wafer/Ta (5 nm)/Cu (200 nm)/CoPt (15–20 nm)] (Tabakovic et al., 2016). Also the coercivity of H_C,//_ = 629 Oe and H_C,⊥_ = 1,220 Oe in Co_80_Pt_20_ films deposited on Ru substrate [oxidized Si wafer/Ta (5 nm)/Ru (200 nm)/CoPt (15–20 nm)] was reported. It is well known that perpendicular anisotropy of CoPt films obtained either by electrodeposition or vacuum deposition highly depend on the underlayer types such as Cu and Ru (Wierman et al., 2002; Pattanaik et al., 2006; Vokoun et al., 2006; Wodarz et al., 2016). Because, in this study, brass substrate was used and parallel coercivities ranged from ∼1,400 to ∼1,600 Oe and perpendicular coercivities ranged from ∼2,200 to ∼2,800 Oe were obtained, we can suggest that the type of substrate strongly affects coercivities of CoPt films.

Figure 3 shows the dependence of current efficiency on H_2_PtCl_6_ concentration and solution pH in the baths. Current efficiency decreased from 22 to 17% with increasing H_2_PtCl_6_ concentration. No work for the dependence of the change of H_2_PtCl_6_ concentration on current efficiency in CoPt alloy was reported as far as we know. Current efficiency as a function of CoSO_4_ concentration (0.1 and 0.25 M) in the bath for electrodeposited CoPt films was measured by Dragos-Pinzaru et al. (Dragos-Pinzaru et al., 2017). They reported that the bath with higher concentration of 0.25 M CoSO_4_ exhibits the current efficiency of about 61–65%, while the bath with lower concentration of 0.1 M CoSO_4_ shows the current efficiency of about 55–60%. The change of CoSO_4_ concentration in the bath resulted in the decrease of about 6% in current efficiency. However, it was reported that deposit Co content in CoPt films was almost the same as 66.9 wt% (87 at%) for both concentrations of 0.1 and 0.25 M CoSO_4_ in the bath. Therefore, some decrease of current efficiency from 22 to 17% with increasing H_2_PtCl_6_ concentration in this study is expected. Because the increase of Co^2+^ concentration in the bath (see Figure 1) from 0 to 0.1 M results in the decrease of deposit Pt content and the increase of deposit Co content in the CoPt films, we can suggest that more Co^2+^ concentration in the bath means more deposit Co content in CoPt films; more H_2_PtCl_6_ concentration in the bath gives more deposit Pt content in the CoPt films, resulting in the decrease of current efficiency.

The dependence of current efficiency on solution pH is also shown in Figure 3. Maximum current efficiency (∼20%) was obtained at pH 9. Dragos-Pinzaru et al. investigated the influence of solution pH (2.5 and 5.5) on the current efficiency of electrodeposited CoPt films from hexachloroplatinate solutions (Dragos-Pinzaru et al., 2017). They reported that the current efficiency in the bath with pH 2.5 and 5.5 was measured to be about 62 and 66%, respectively. We believe from Figure 1 that about three times higher current efficiency of CoPt films reported by Dragos-Pinzaru et al. (Dragos-Pinzaru et al., 2017) than that of this study may be attributed to the different chemical compositions in the baths. The change of solution pH somewhat has an influence on the current efficiency. The current efficiency highly depends on the chemical compositions in the baths rather than solution pH.

Figure 4 shows the dependence of coercivity and squareness of binary CoPt thin film alloys on H_2_PtCl_6_ concentration. This experimental work was carried out in order to find higher coercivity of CoPt films in the bath compositions and operating conditions as shown in Table 1. The optimum coercivity (H_C,//_ = ∼1,000 Oe and H_C,⊥_ = ∼2,700 Oe) was obtained at 0.01 M Pt concentration, while parallel and perpendicular squarenesses were measured as ∼0.18 and ∼0.3, respectively.

Figure 5 shows the dependence of coercivity and squareness of CoPt alloys on solution pH. The optimum coercivity (H_C,//_ = ∼1,250 Oe and H_C,⊥_ = ∼1,300 Oe) was obtained at solution pH 8–9. Parallel and perpendicular squarenesses at solution pH 8–9 were measured as ∼0.6 and ∼0.1, respectively. It was observed that the change of solution pH has a considerable effect on the coercivity of CoPt films.

Figure 6 exhibits the dependence of current efficiency of binary CoPt and ternary CoPtP electrodeposits on current density. The current efficiencies were slightly decreased with increasing current density for both CoPt and CoPtP electrodeposits. The addition of 0.1 M NaH_2_PO_2_ in the plating bath resulted in the slight decrease of current efficiency from 10 to 7%. Dragos-Pinzaru et al. investigated the influence of saccharin (with/without 3.89 mM) as an additive on current efficiency of CoPt films electrodeposited from hexachloroplatinate solutions (Dragos-Pinzaru et al., 2017). Higher current efficiency (78%) was observed in CoPt films electrodeposited from the bath containing no saccharin, while CoPt films electrodeposited from the bath containing 3.89 mM saccharin exhibits lower current efficiency (66%). Therefore, we can suggest that the current efficiency obtained in CoPt films electrodeposited from hexachloroplatinate solutions was influenced by the type of additive such as saccharin (Dragos-Pinzaru et al., 2017) or sodium hypophosphite (NaH_2_PO_2_) (this study). In this study, the current efficiency was measured as 22% at the most or less. Therefore, we can summarize from Figures 1, 3, 6 that current efficiency obtained in CoPt films electrodeposited from hexachloroplatinate solutions much depend on the bath compositions rather than the type of additives, solution pH, CoSO_4_, and H_2_PtCl_6_. That is, current efficiencies in the baths used by Dragos-Pinzaru et al. (Dragos-Pinzaru et al., 2017) were much higher than that in the baths used by this study.

Figures 7, 8 show the corresponding coercivity and squareness of binary CoPt (Figure 7; electrodeposited from the bath containing no NaH_2_PO_2_) and ternary CoPtP electrodeposits (Figure 8; electrodeposited from bath containing 0.1 M NaH_2_PO_2_). Figures 7, 8 were carried out at optimum conditions (using the conditions from Table 1; Co^2+^ and Pt^4+^ concentrations, solution pH, and current density) in order to get the best coercivity in CoPt films. In binary CoPt electrodeposits, high coercivity (H_C,//_ = ∼1,600 Oe and H_C,⊥_ = ∼2,100 Oe) was obtained at the current density of 7.5 mA/cm^2^. On the other hand, parallel and perpendicular squarenesses at the current density of 7.5 mA/cm^2^ were measured as ∼0.35 and ∼0.2, respectively. There is no research work for the effect of adding sodium hypophosphite (NaH_2_PO_2_) into the baths for binary CoPt thin films. In ternary CoPtP electrodeposits of this study (Figure 8), high coercivity (H_C,//_ = ∼3,000 Oe and H_C,⊥_ = ∼3,500 Oe) was obtained from 7.5 to 20 mA/cm^2^. Also, parallel and perpendicular squarenesses at the current density of 7.5–20 mA/cm^2^ were measured as ∼0.4 and 0.2–0.4, respectively.

Figure 9 shows the dependence of coercivity and squareness of CoPtP alloys on NaH_2_PO_2_ concentration in the plating baths. Parallel coercivity increased from ∼1,700 to ∼2,700 Oe with increasing NaH_2_PO_2_ concentration from 0.01 to 0.8 M, while perpendicular coercivity was maintained at the range of 3,000–3,500 Oe. Perpendicular squareness was slightly increased and parallel squareness was increased from ∼0.2 to 0.37 with increasing NaH_2_PO_2_ concentration. The addition effect of NaH_2_PO_2_ concentration in the bath for electrodeposited CoPt films was much higher in parallel coercivity than in perpendicular coercivity. Also, the similar effect both for parallel and perpendicular squarness was observed.

**FIGURE 10:**
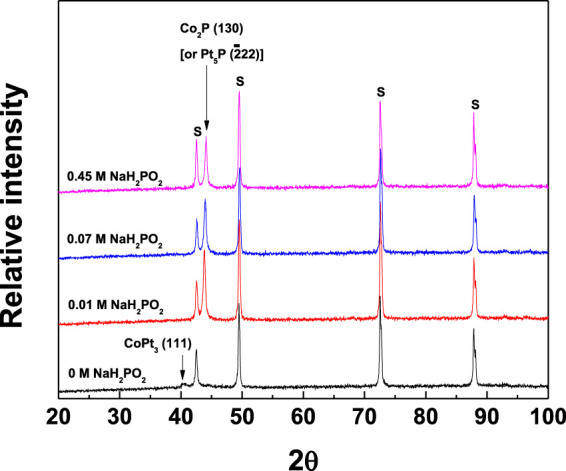
XRD patterns of CoPt and CoPtP thin films electrodeposited on brass substrate with the change of NaH_2_PO_2_ concentration in the plating baths; relative intensity vs NaH_2_PO_2_ concentration (S: substrate).

Figure 10 shows XRD patterns of binary CoPt and CoPtP thin film alloys with increasing NaH_2_PO_2_ concentration in the baths. For the binary CoPt thin film, it is analyzed that CoPt thin films consist of mainly amorphous crystalline and small intensity of CoPt_3_ (111) phases (JCPDS file #: 29–499). For the ternary CoPtP thin film, CoPtP thin films consist of both amorphous crystalline and Co_2_P (130) (JCPDS file #: 6–306) [or Pt_5_P_2_ ($\bar{2}22$) (JCPDS file #: 23–465)] peaks. The addition of NaH_2_PO_2_ into the baths for the binary CoPt films results in the considerable increase of intensity of Co_2_P (130) (JCPDS file #: 6–306) [or Pt_5_P_2_ ($\bar{2}22$) (JCPDS file #: 23–465)] peaks. Tabakovic et al. investigated the influence of different substrates (Cu and Ru) on the peaks in XRD patterns in CoPt films electrodeposited from hexachloroplatinate solutions (Tabakovic et al., 2016). Zana et al. also reported the same results of XRD using electrodeposited CoPt on Cu seed layer (Zana and Zangari, 2004). It was reported that CoPt films electrodeposited on Cu substrate [oxidized Si wafer/Ta (5 nm)/Cu (200 nm)/CoPt (15–20 nm)] consists of hcp CoPt (10.0), (00.2) and (10.1), at 41.2°, 43.4°, 46.4° 2θ values, respectively (Tabakovic et al., 2016). On the other hand, CoPt films electrodeposited on Ru substrate [oxidized Si wafer/Ta (5 nm)/Ru (200 nm)/CoPt (15–20 nm)] exhibits hcp CoPt (10.0) and (00.2) at 41.2° and 43.4° 2θ values, respectively (Tabakovic et al., 2016).

It was reported that Ru underlayer results in both the improvement of the microstructure [absence of hcp CoPt (00.2)] and enhancement of perpendicular anisotropy (H_C,//_= 629 Oe and H_C,⊥_ = 1,220 Oe) in CoPt films (Tabakovic et al., 2016). The same results were observed in CoPt films obtained either by vacuum deposition or electrodeposition (Tabakovic et al., 2016; Pattanaik et al., 2006; Wodarz et al., 2016; Wierman et al., 2002; Vokoun et al., 2006). Also, Dragos-Pinzaru et al. reported that very high perpendicular coercivity (H_C,⊥_ = 4,500–6,700 Oe) in CoPt films is attributed to both Ru substrate and thickness (10–30 nm) of CoPt film (Dragos-Pinzaru et al., 2017). They suggested that high perpendicular coercivity of Co_71_Pt_21_ film (∼15 nm thickness) deposited on Ru seed layer is resulted from the addition of saccharin in the baths, resulting in the dramatic improvement of hcp (00.2) crystal structure. However, in this study, the binary CoPt film electrodeposited on brass substrate mainly consists of nanocrystalline with only a very small intensity of CoPt_3_ (111) peak (JCPDS file #: 29–499). On the other hand, the ternary CoPtP film electrodeposited in the baths containing NaH_2_PO_2_ showed the dramatic improvement of Co_2_P (130) [or Pt_5_P_2_ ($\bar{2}22$)] peaks, resulting in the increase of parallel coercivity from ∼1,700 Oe to ∼3,000 Oe and the increase of perpendicular coercivity from ∼3,200 Oe to ∼3,700 Oe. These results in this study may be attributed to the different substrate (brass) and the different bath compositions compared to previous papers reported by another researcher (Tabakovic et al., 2016; Dragos-Pinzaru et al., 2017). Some important results in CoPt/CoPtP films (for high coercivities) were tabulated in Table 3 to compare each other.

**TABLE 3:** Comparison of some important results representing high coercivities in CoPt/CoPtP thin films.

Coercivity (Oe)		Alloy/substrate	References
H_c,//_	H_c,⊥_		
629	1,220	CoPt/Ru	Tabakovic et al. (2016)
221	254	CoPt/Cu	
—	4,500–6,700	CoPt (10–30 nm thick)/Ru	Dragos-Pinzaru et al. (2017)
1700–3,000	3,200–3,700	CoPt/brass	This study

In summary, it is believed that the coercivity and XRD patterns [Figures 9, 10] in this study are affected not only by the type of substrate but also by chemical composition and operating conditions in the baths for electrodeposition. Although there is neither SEM image nor optical microscope image in this study, the smooth, bright and shiny surfaces of all the CoPt and CoPtP thin films were observed.

## Conclusion

Magnetic CoPt and CoPtP thin film alloys were fabricated by electrodeposition process from the baths containing chloroplatinic acid, cobalt pyrophosphate and sodium hypophosphite. Influence of several electrodeposition parameters such as solution compositions (e.g [Pt^4+^] [Co^2+^] and [NaH_2_PO_2_]), solution pH, current density on current efficiency, magnetic properties, and microstructure was systematically investigated. It is believed that relatively low current efficiency in this article compared to that reported by Dragos-Pinzaru et al. (Dragos-Pinzaru et al., 2017) may be attributed to the different chemical compositions because of much more current consumption in the evolution of side reactions in this study. Parallel coercivities ranged from ∼1,400 to ∼1,600 Oe and perpendicular coercivities ranging from ∼2,200 to ∼2,800 Oe for binary CoPt films in this article were obtained. It is believed that the type of substrate strongly affects coercivities of CoPt films. Also, more Co-pyrophosphate concentration in the bath results in more deposit Co content in CoPt films; more H_2_PtCl_6_ concentration in the bath more deposit Pt content. Current efficiency obtained in CoPt films electrodeposited from hexachloroplatinate solutions much depend on the bath compositions rather than the type of additives, solution pH, CoSO_4_, and H_2_PtCl_6_. In summary, the XRD patterns and coercivity in this study are affected by both the type of substrates and chemical composition and operating conditions in the baths for electrodeposition.

## Data Availability

The original contributions presented in the study are included in the article/Supplementary Material, further inquiries can be directed to the corresponding author.
