# Supplementary material for: Magnetic Properties of Electrodeposited Cobalt-Platinum (CoPt) and Cobalt-Platinum-Phosphide (CoPtP) Thin Films
Source: Front Chem. 2021 Sep 10;9:733383. doi: 10.3389/fchem.2021.733383 (PMC8462268; doi:10.3389/fchem.2021.733383)
Supplement: Supplementary file 1 [file DataSheet1.PDF]

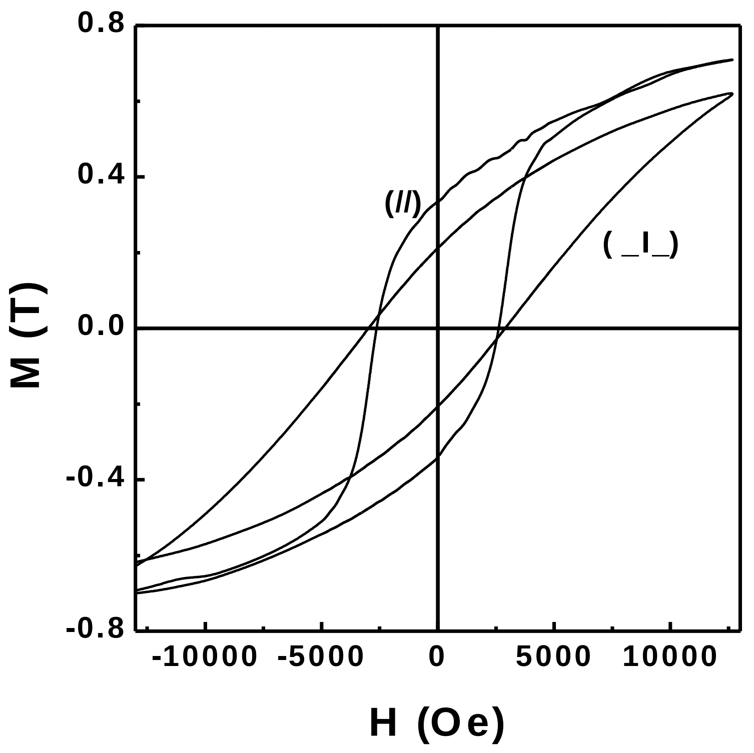

One of B (or M)-H data in Figure 9

Hysteresis loops of electrodeposited CoPtP thin film at 5 mA cm^{-2}

Solution: 0.02 M $Na_2Co(P_2O_7)_2$ + 0.01 M H_2PtCl_6 + 0.1 M NaH_2PO_2 + 0.365 M Na_3PO_4
